# Explainable and Robust Deep Learning for Liver Segmentation Through U-Net Network

**DOI:** 10.3390/diagnostics15070878

**Published:** 2025-03-31

**Authors:** Maria Chiara Brunese, Aldo Rocca, Antonella Santone, Mario Cesarelli, Luca Brunese, Francesco Mercaldo

**Affiliations:** 1 Department of Medicine and Health Sciences “Vincenzo Tiberio”, University of Molise, 86100 Campobasso, Italyantonella.santone@unimol.it (A.S.);; 2 Department of Engineering, University of Sannio, 82100 Benevento, Italy; mcesarelli@unisannio.it

**Keywords:** liver, U-Net, segmentation, biomedical images, bioimages, deep learning, explainability, robustness

## Abstract

**Background/Objectives:** Clinical imaging techniques, such as magnetic resonance imaging and computed tomography, play a vital role in supporting clinicians by aiding disease diagnosis and facilitating the planning of appropriate interventions. This is particularly important in malignant conditions like hepatocellular carcinoma, where accurate image segmentation, delineating the liver and tumor, is a critical initial step in optimizing diagnosis, staging, and treatment planning, including interventions like transplantation, surgical resection, radiotherapy, portal vein embolization, and other procedures. Therefore, effective segmentation methods can significantly influence both diagnostic accuracy and treatment outcomes. **Method:** In this paper, we propose a deep learning-based approach aimed at accurately segmenting the liver in medical images, thus addressing a critical need in hepatic disease diagnosis and treatment planning. We consider a U-Net architecture with residual connections to capture fine-grained anatomical details. We also take into account the prediction explainability, aiming to highlight, in the image under analysis, the areas that are symptomatic for a certain segmentation. In detail, by exploiting the U-Net architecture, two different models are trained with two annotated datasets of computed tomography medical images, resulting in four different experiments. **Results:** We consider two different datasets to improve robustness and generalization across diverse patient populations and imaging conditions. Experimental results demonstrate that the proposed method obtains interesting performances, with an accuracy ranging from 0.81 to 0.93. **Conclusions:** We thus show that the proposed method can provide a reliable and efficient solution for automated liver segmentation, promising significant advancements in clinical workflows and precision medicine.

## 1. Introduction

The liver, a vital organ responsible for numerous essential functions such as detoxification, protein synthesis, and the production of biochemicals necessary for digestion, plays a critical role in maintaining overall health [1,2]. This is the reason why accurate liver segmentation, the process of delineating the liver and its structures within medical imaging [3,4], has become increasingly important in medical diagnostics and treatment planning. This advanced imaging technique allows for precise identification and analysis of liver anatomy and pathology, aiding in the diagnosis of liver diseases, assessment of tumor growth, and planning of surgical interventions [5,6]. As medical imaging technology continues to evolve, liver segmentation is crucial, offering enhanced precision in disease monitoring, facilitating targeted therapies, and ultimately improving patient outcomes [7,8]. Understanding the significance of liver segmentation underscores its impact on the quality and effectiveness of healthcare, particularly in the management of hepatic conditions [9].

As a matter of fact, liver segmentation can be helpful in several medical scenarios, for instance, accurate segmentation helps in identifying and characterizing liver tumors, essential for diagnosing liver cancer [3,10]. Moreover, detailed segmentation assists surgeons in planning the removal of liver tumors or damaged tissue while preserving as much healthy liver as possible. Furthermore, precise liver segmentation ensures that radiation is accurately targeted to cancerous tissues, minimizing damage to healthy liver tissue [11,12]. Another context where segmentation is helpful is due to their ability to monitor changes in liver size and shape, helping to track the progression of chronic liver diseases. It also allows for the assessment of how well a patient is responding to treatments such as chemotherapy or antiviral therapy [13]. From the perspective of the development of computational models, segmented liver images are used to create models that can predict disease progression and treatment outcomes and can also support the development of new therapies and diagnostic tools by providing detailed anatomical and pathological data. Segmentation enables precise volume measurements of the liver and its lesions, which are crucial for assessing the severity of diseases and planning treatments, and by integrating with other imaging modalities, segmentation helps in evaluating liver function and perfusion [14,15,16].

Overall, liver segmentation enhances the accuracy and effectiveness of medical diagnostics, treatment planning, and disease monitoring, significantly improving patient care and outcomes [17,18,19].

Recently, computer vision (CV) has been emerging as a subfield of artificial intelligence that deals with the interpretation and understanding of visual information from the real world. In particular, segmentation is a fundamental task within computer vision, involving the partitioning of an image into meaningful regions or objects [17,20,21].

As a matter of fact, CV can significantly enhance liver segmentation by automating the detection and delineation of liver boundaries in medical images, such as computed tomography (CT). As a matter of fact, it can reduce the variability and errors associated with manual segmentation, improving accuracy and efficiency in clinical workflows. Through the use of deep learning models, CV algorithms can learn to identify complex patterns and structures in liver tissues, enabling precise segmentation even in cases with challenging anatomical variations. Thus, CV allows for faster and more consistent segmentation, facilitating better diagnosis, treatment planning, and monitoring of liver diseases [22,23,24].

Starting from these considerations, in this paper, we propose a method aimed at performing automatic liver segmentation by exploiting CV, with particular regard to deep learning. The proposed method also takes into account explainability, with the aim of highlighting the areas on the medical images that are related to a certain prediction. From the model point of view, we exploit the U-Net model, a type of convolutional neural network primarily designed for biomedical image segmentation. It uses an encoder–decoder architecture, where the encoder captures spatial features and the decoder reconstructs the image, allowing precise localization. A feature of U-Net is its skip connections, which help retain spatial information by transferring feature maps from the encoder to corresponding layers in the decoder. In order to take into account the explainability (i.e., a way to enable human users to understand and trust the results and outputs of a machine learning model), we resort to Gradient-weighted Class Activation Mapping (i.e., Grad-CAM) [17,25,26,27]. Grad-CAM is a visualization technique used to interpret the predictions of convolutional neural networks. It basically works by highlighting the important regions in an input image that influence the decision of the model. It works by computing the gradients of a target class score with respect to the feature maps in the final convolutional layer and using these gradients to produce a heatmap of class-discriminative regions. This method is particularly useful for understanding model behavior in tasks like image classification, object detection, and medical imaging [17,20,28,29].

With the aim of demonstrating the effectiveness of the proposed method, we resort to two different datasets, both of them freely available for research purposes; in particular, we provide, in the experimental analysis section, four different experiments, the first one by exploiting the model built with the first dataset and evaluated with (not used in the training) images with the first dataset, the second one with the same model of the first experiment that is evaluated with images belonging to the second dataset. The third experiment considers the second dataset for model building and (not used in the training) model testing images, while the last experiment is related to the model of the third experiment evaluated with images belonging to the first dataset. The idea of these four experiments (in particular, the second and the fourth ones, where images belonging from different datasets are exploited for training and testing) is to evaluate the model robustness with different images, acquired from different machinery, with different conditions and from different human operators, exploited for training and testing purposes.

This study aims to investigate the effectiveness, robustness, and interpretability of deep learning-based liver segmentation. Specifically, we address the following research questions:

RQ1: How effectively can a deep learning-based U-Net model with residual connections improve the accuracy of liver segmentation in CT images compared to traditional methods?

RQ2: How does the integration of Grad-CAM enhance the explainability of segmentation predictions, making them more interpretable for clinical applications?

RQ3: Can the proposed model generalize well across different datasets acquired from diverse imaging conditions, scanner types, and patient populations?

Below, we itemize the main contributions of the proposed method:

- Development of an Explainable Liver Segmentation Model: We propose a U-Net-based deep learning model incorporating residual connections to improve feature propagation and segmentation accuracy.
- Integration of Explainability with Grad-CAM: Unlike conventional black-box deep learning models, our approach enhances transparency by employing Grad-CAM to visualize and interpret segmentation decisions.
- Robustness Across Multiple Datasets: The model is trained and evaluated on two different publicly available CT liver segmentation datasets, ensuring improved generalization across imaging conditions, scanner types, and patient populations.
- Comprehensive Experimental Validation: We conduct extensive experiments across four different scenarios, evaluating the model’s segmentation accuracy, Dice coefficient, and Intersection over Union (IoU) to assess its robustness and effectiveness.
- Potential for Clinical Application: By improving segmentation accuracy while offering explainability, the proposed method enhances trust in AI-driven liver segmentation, making it more suitable for real-world clinical workflows, including preoperative planning and disease monitoring.

The paper proceeds as follows. In Section 2, we provide preliminary notions related to the U-Net architecture considered by the proposed method, with the aim of making the paper self-contained; Section 3 depicts in detail the proposed method for explainable automatic liver segmentation; the experimental analysis aimed to show the performances, and the robustness of the proposed method is presented in Section 4. An overview of the state-of-the-art research in the context of liver segmentation is shown in Section 5, and finally, the conclusion and future research plans are presented in Section 6.

## 2. Background

In this section, we provide preliminary notions about the deep learning model we considered for liver segmentation, with the aim of making the paper self-contained.

Deep learning offers a variety of architectures for medical image segmentation, with Convolutional Neural Networks (CNNs) being the foundation of most. Among these, U-Net stands out as a specialized architecture tailored for biomedical imaging tasks, including segmentation. Its name derives from its distinctive “U” shape, which reflects its symmetric encoder–decoder design. This architecture is particularly well-suited for tasks that demand accurate localization and identification of specific image regions.

The U-Net architecture consists of two primary components: the contracting path, also known as the encoder, and the expansive path, referred to as the decoder. These paths are connected by a bottleneck, forming the central bridge. Together, these components enable the model to learn hierarchical feature representations while retaining spatial details essential for segmentation.

The contracting path is responsible for extracting high-level features and capturing the broader context of the input image. This path comprises several stages, each consisting of two convolutional layers followed by a ReLU activation function and a max-pooling operation. The convolutional layers use small 3 × 3 kernels to extract features, while the max-pooling layers reduce the spatial dimensions by half, thereby increasing the receptive field and enabling the network to learn more abstract features. This process is repeated multiple times, progressively reducing the spatial dimensions and deepening the feature representations.

At the center of the U-Net architecture lies the bottleneck, which serves as a connection between the encoder and the decoder. This segment includes two 3 × 3 convolutional layers with ReLU activations. Unlike the contracting path, the bottleneck does not employ max-pooling layers, preserving the spatial resolution of the feature maps. This design ensures that the most critical features are retained for reconstruction.

The expansive path, or decoder, is tasked with reconstructing the segmentation map by progressively upsampling the feature maps. Each stage of the decoder begins with a transposed convolution, also known as up-convolution, which doubles the spatial dimensions of the feature maps. The upsampled features are then concatenated with the corresponding features from the contracting path through skip connections. These skip connections play a pivotal role in preserving spatial information and finer details that might have been lost during downsampling. Following concatenation, two 3 × 3 convolutional layers with ReLU activations are applied to refine the features and prepare them for the next upsampling stage.

The final layer of the U-Net architecture is a 1 × 1 convolutional layer that reduces the feature map to the desired number of output classes. For binary segmentation tasks, this layer typically outputs two classes, representing the segmented region and the background. The resulting map is further processed with a sigmoid activation function to produce the final segmentation output.

The U-Net architecture’s design, combining hierarchical feature extraction with spatial detail retention, makes it an ideal choice for medical image segmentation. Its ability to deliver accurate and precise segmentation maps has made it a cornerstone in biomedical imaging applications.

This architecture allows U-Net to effectively capture both global context and fine details, making it highly effective for various segmentation tasks, particularly in medical imaging.

In the following, we motivate the reason why we resort to this specific model for liver segmentation, by discussing the several advantages offered by U-Net over traditional segmentation methods, particularly in the context of biomedical imaging.

First, U-Net supports end-to-end learning, training directly on raw image data and corresponding segmentation masks. This enables it to automatically learn features and representations from the data, optimizing for the specific task and reducing the need for manual feature engineering. In contrast, traditional methods often rely on manual feature extraction, which can be time-consuming and prone to human error.

Second, U-Net efficiently handles small datasets by utilizing data augmentation techniques like rotations, shifts, and flips. It leverages symmetry and skip connections, making the most out of limited training data. On the other hand, traditional methods are less capable of handling small datasets without overfitting and often require extensive manual tuning and augmentation strategies.

Third, U-Net excels at precision and accuracy, particularly in localizing and segmenting structures of varying sizes. The use of skip connections allows it to retain fine-grained spatial information, leading to more precise segmentations. Traditional methods may struggle with small or complex structures and often rely on heuristic or rule-based approaches, which can be less precise.

Fourth, U-Net offers flexibility and adaptability, making it versatile enough to be applied to a wide range of segmentation tasks, beyond medical imaging, such as satellite imagery and natural scene segmentation. It is also easy to modify and extend with additional layers or modules to suit specific needs. In contrast, traditional methods are often task-specific, requiring significant redesign and retraining for different segmentation tasks, and are less flexible in adapting to new types of data or changing requirements.

Fifth, U-Net demonstrates efficiency in inference, capable of real-time or near-real-time processing, which is ideal for applications like intraoperative guidance. It is well suited for GPU acceleration, significantly speeding up processing times. Traditional methods tend to be slower, especially when complex pre-processing and feature extraction steps are involved, and may not be optimized for parallel processing or hardware acceleration.

Sixth, U-Net effectively integrates contextual information, capturing both local and global context, which is essential for understanding complex structures in medical images. By combining high-level semantic information with low-level details through its expansive path and skip connections, it achieves a deeper understanding of the data. Traditional methods, however, often lack the ability to integrate multi-scale contextual information, requiring separate modules or steps to handle different scales, thereby increasing complexity.

Seventh, U-Net reduces the annotation effort by providing high-quality initial segmentations that can be fine-tuned by experts. This minimizes the time and effort needed for creating large annotated datasets. In comparison, traditional methods typically demand extensive manual annotation and correction, which can be labor-intensive and time-consuming, with segmentations often requiring significant post-processing and refinement.

Lastly, U-Net demonstrates strong generalization capabilities, performing well across different datasets and imaging modalities. Thanks to its deep learning foundations, it can handle variations in image quality, noise, and artifacts. In contrast, traditional methods are often sensitive to variations in imaging conditions and may require separate tuning for different datasets, with generalization capabilities limited without extensive customization and parameter adjustment.

## 3. Materials and Methods

**Figure 1:**
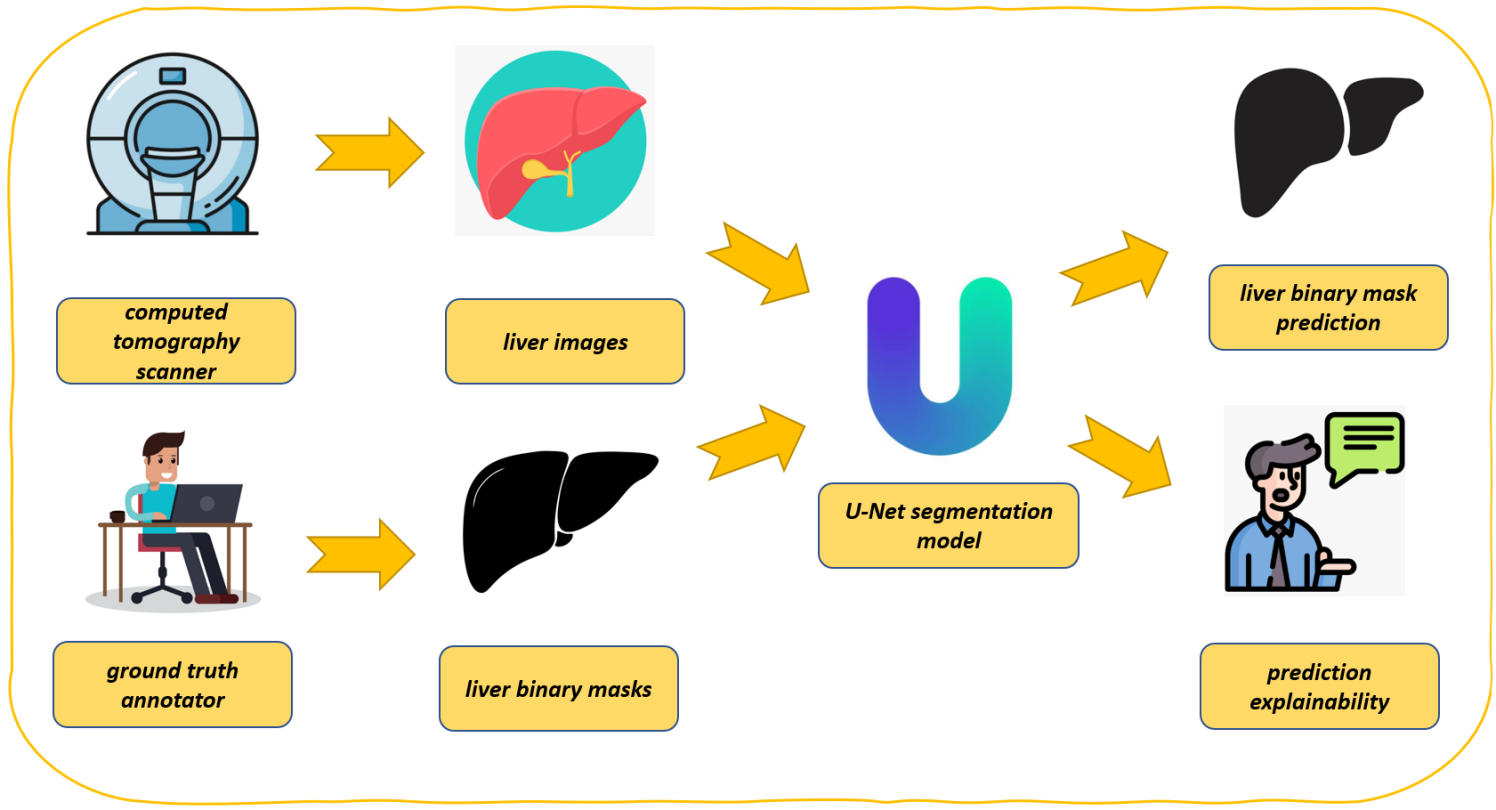
The workflow related to the proposed method for explainable liver segmentation.

In this section, we describe the proposed method for explainable liver segmentation, whose workflow is shown in Figure 1. As introduced in the previous section for liver segmentation, we resort to the U-Net model, enhanced with explainability through Grad-CAM. We also take into account model robustness by considering two different datasets in the experimental analysis.

Figure 1 presents the proposed workflow for automated liver segmentation from CT images, utilizing a U-Net deep learning model alongside explainability techniques provided by Grad-CAM.

As shown in Figure 1, the proposed method is composed of several steps, explained in detail below.

- The process begins with the acquisition of medical imaging data (i.e., computer tomography scanner in Figure 1). CT scanners capture detailed cross-sectional images of the abdomen, which include the liver. These images are essential for constructing accurate 3D representations of the liver. The CT scanner indicates the source of the initial input data.
- The CT scans, shown as liver images, are extracted from the abdominal region (i.e., liver images in Figure 1). These images serve as the raw input for the segmentation task. Each image is typically a 2D slice of the liver, which is part of a larger 3D dataset.
- Before training the model, the CT images must be annotated to create a reference standard (i.e., ground truth annotator in Figure 1). Expert radiologists or medical professionals manually outline the liver boundaries in each image, generating accurate segmentation masks. These annotations are represented as liver binary masks in the figure, where pixels corresponding to the liver are marked as 1 (or white) and all other pixels as 0 (or black). The annotator is symbolized by a person working at a computer, highlighting the manual effort involved in creating these ground truth labels.
- These masks (i.e., liver binary masks in Figure 1) are critical for supervised learning, as they provide the U-Net model with examples of correct segmentations, against which its predictions can be compared during training.
- The U-Net model, depicted at the center of the figure, is the core of the proposed method (i.e., U-net segmentation model in Figure 1). U-Net is a CNN specifically designed for biomedical image segmentation tasks. It features a unique architecture with a contracting path (encoder) that captures context and a symmetric expanding path (decoder) that enables precise localization. The liver images and corresponding binary masks are fed into the U-Net model during training. The model learns to map input images to their respective segmentation masks, effectively learning to identify and delineate the liver from the surrounding tissues.
- After training, the U-Net model is capable of segmenting the liver in unseen CT images (i.e., liver binary mask prediction in Figure 1). Given a new image, the model outputs a predicted binary mask, which highlights the liver region. This predicted mask is intended to replicate the accuracy of the ground truth annotations, allowing for automated liver segmentation. The figure shows this as a black-and-white liver shape, emphasizing the binary nature of the output.
- To ensure that the model predictions are reliable and understandable (i.e., prediction explainability in Figure 1), the proposed method incorporates explainability through Grad-CAM. In a nutshell, Grad-CAM generates a heatmap that overlays on the input image, showing which regions the U-Net model focused on while making its prediction. This allows practitioners to verify that the model is basing its decisions on relevant anatomical features, such as the liver’s edges, rather than irrelevant background areas. The explainability aspect is depicted by an individual explaining or discussing the model’s predictions, symbolizing the interpretability and validation process.

As shown in Figure 1, the proposed method represents a process where CT images are annotated by medical domain experts to create ground truth masks. These masks are used to train a U-Net model that learns to segment the liver automatically. The model’s predictions are subsequently explained and validated using Grad-CAM, ensuring that the segmentation is not only accurate but also interpretable, with the aim of boosting the adoption of deep learning in the real-world medical domain.

The use of U-Net ensures high accuracy in segmentation, while Grad-CAM addresses the need for transparency in computational intelligence-driven medical applications. This integration of segmentation and explainability forms a robust approach to liver segmentation, suitable for clinical use where both performance and interpretability are critical.

The use of Grad-CAM for explainability in segmentation tasks might not be immediately apparent, as it is traditionally employed to visualize class-relevant activations in classification networks. Nevertheless, its application in segmentation tasks offers several practical advantages and serves distinct purposes.

Firstly, Grad-CAM provides a means to visualize model attention, helping to identify the regions of an image the segmentation model focuses on when making decisions. This is especially valuable for verifying whether the model is leveraging the correct image features to segment the regions of interest. Importantly, while Grad-CAM does not enhance model performance, it offers a visual representation of the areas that the model considers significant during analysis.

Secondly, Grad-CAM contributes to debugging and interpretability, which are essential in critical domains such as medical segmentation or autonomous driving. By highlighting the specific parts of the image influencing predictions, it enables researchers and engineers to pinpoint and address potential errors in the model’s behavior.

Additionally, Grad-CAM serves as a tool for model validation, ensuring that the activated regions align with expectations. For instance, in an organ segmentation task like the liver segmentation addressed in this paper, Grad-CAM can confirm whether the model focuses on the appropriate organs during the segmentation process.

Finally, it aids in feature analysis by revealing the features the model is learning. This insight can guide further refinement of the model or the input data, ultimately contributing to improved overall performance.

To integrate Grad-CAM into the proposed segmentation task, the process involves selecting an intermediate convolutional layer within the U-Net architecture. The activations of this layer are computed, along with the gradients of the loss function with respect to these activations. These gradients are then used to weight the activations, which are subsequently combined to produce a heatmap. Finally, this heatmap is visualized in conjunction with the segmentation prediction to provide insights into the model’s focus areas.

Using Grad-CAM in segmentation tasks proves to be a valuable strategy for improving the interpretability, debugging, and validation of segmentation models. Although originally developed for classification, adapting Grad-CAM to segmentation contexts offers meaningful insights into the model’s decision-making process and highlights potential areas for further refinement.

To evaluate the performance of the proposed method for explainable liver segmentation, we resort to Accuracy (by comparing the predicted segmentation mask with the ground truth mask and calculating the proportion of correctly classified pixels), Dice and IoU metrics.

The model was developed from authors by exploiting the Python programming language (3.9.19 version).

To evaluate the effectiveness of the proposed method, two different datasets are exploited, both of them retrieved from the Kaggle website and freely available for research purposes (and thus for replication purposes). The first one is the CT liver dataset https://www.kaggle.com/datasets/zxcv2022/digital-medical-images-for–download-resource, accessed on 27 February 2025 (i.e., the DI dataset), while the second one is the Segmentation of Liver https://www.kaggle.com/datasets/priyamsaha17/segmentation-of-liver dataset, accessed on 27 February 2025 (i.e., the DII dataset). The DI dataset is composed of 116 CT liver images with the related binary mask; in particular, 96 CT images are considered for training and the remaining 20 as the testing dataset. The DII dataset is composed of 282 CT liver images with the related binary mask, and in this case as well, 20 CT liver images are exploited as the testing dataset and the remaining 262 CT liver images are considered as the training dataset.

## 4. Results

In this section we describe the results of the experimental analysis. First of all, we describe in detail the exploited datasets.

### 4.1. Image Resolution and Format

- The images in both datasets are provided in grayscale with a resolution of 512 × 512 pixels, ensuring a high level of anatomical detail necessary for accurate segmentation.
- All images are stored in DICOM (Digital Imaging and Communications in Medicine) format, which preserves essential metadata such as acquisition parameters and patient positioning.

### 4.2. Number of Images and Data Distribution

- DI Dataset: Contains 116 CT liver scans with corresponding binary segmentation masks. The dataset is split into 96 images for training and 20 for testing to maintain a balanced evaluation.
- DII Dataset: Includes 282 CT liver images, where 262 are used for training and 20 for testing to assess the model’s generalizability.

### 4.3. Patient Diversity and Imaging Conditions

- The datasets consist of CT scans from multiple patients, captured using different CT scanners under varied acquisition settings (contrast-enhanced and non-contrast images).
- This diversity ensures the dataset reflects real-world variability in patient anatomy, liver pathology, scanner resolution, and image noise levels, which is crucial for evaluating model robustness.

### 4.4. Preprocessing

To ensure consistency across both datasets and enhance the training process, the following preprocessing steps were applied:

#### 4.4.1. Resizing and Normalization

- Since the original image sizes varied slightly across samples, all images were resized to 256 × 256 pixels to optimize computational efficiency while preserving critical anatomical structures.
- Min-max normalization was applied to scale pixel intensity values between 0 and 1, reducing the impact of intensity variations due to contrast agents or different scanning protocols.

#### 4.4.2. Augmentation for Robustness

Data augmentation techniques were applied to prevent overfitting and improve generalization:

- Rotation ($\pm 20^{{}^{\circ}}$) to account for variations in patient positioning.
- Horizontal and vertical flipping to enhance symmetry recognition.
- Random cropping and zooming (±10%) to simulate variations in scan resolutions.
- Gaussian noise injection to mimic real-world imaging noise.

#### 4.4.3. Segmentation Mask Refinement

- The provided ground truth masks were binarized (liver = 1, background = 0) to standardize segmentation outputs across datasets.
- To correct minor inconsistencies in annotations, morphological operations (erosion and dilation) were applied, improving mask clarity and boundary precision.

By incorporating these preprocessing techniques, we ensure that the deep learning model is trained on a standardized, diverse, and well-balanced dataset, facilitating robust and generalizable liver segmentation.

**Figure 2:**
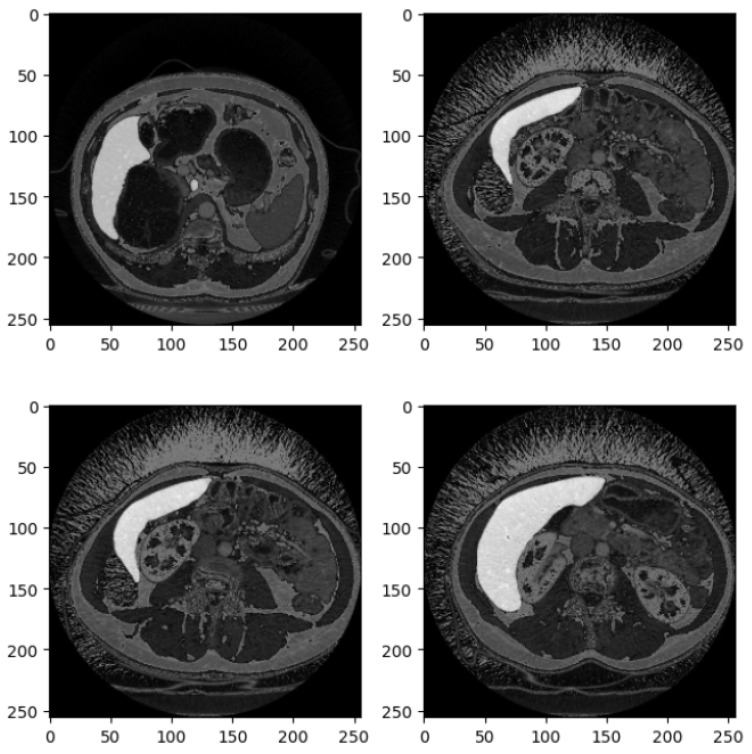
Four examples of CT images with the related mask obtained from the DI dataset.

Figure 2 shows four examples of CT liver images related to the DI dataset.

Figure 2 presents four axial slices from a CT scan, likely of the abdomen. The scans are in grayscale, adhering to standard medical imaging practices. Each image slice is square with dimensions of 250 × 250 pixels, potentially corresponding to a specific magnification level or scale within the imaging modality. These slices represent axial (cross-sectional) views of the abdomen, showcasing various abdominal organs, including the liver, kidneys, stomach, intestines, and possibly sections of the spine and abdominal musculature.

In the top left image, sections of the liver (on the left side) and the kidney (on the right side) are visible, with part of the spinal column centrally located. The top right image depicts a slightly lower section of the abdomen, where the liver occupies a larger portion of the view, and a section of the bowel is observable. The bottom left slice reveals a broader section of the abdominal cavity, with distinct bowel loops that may show gas-filled intestines or contrast material. The bottom right image features the liver more prominently, extending across the section, and includes parts of the gastrointestinal system, possibly the colon.

The images appear to be contrast-enhanced, as indicated by the bright white regions representing areas of high contrast uptake, such as blood vessels or perfused organs. The overall quality of the images is high, with minimal noise, suggesting that they were acquired at an adequate resolution suitable for diagnostic or research purposes. The grayscale intensity is consistent across all slices, indicating uniform windowing and leveling, which is essential for evaluating the radiodensity of the tissues depicted.

**Figure 3:**
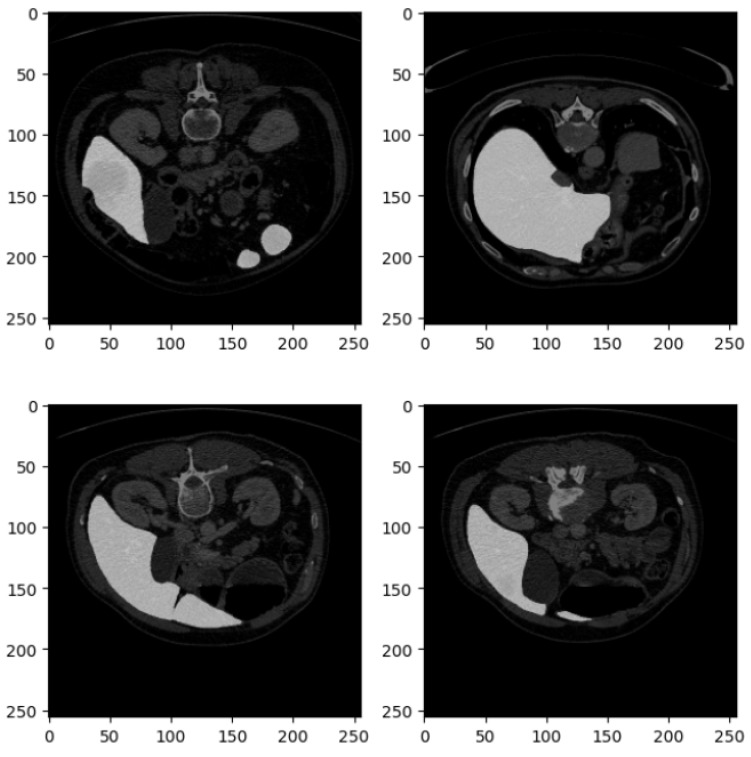
Four examples of CT images with the related mask obtained from the DII dataset.

Figure 3 shows four related examples of CT liver images belonging to the DII dataset.

Figure 3, similar to Figure 2, consists of four axial slices from a CT scan, each displaying different cross-sections of the abdominal region. These images are part of the DII dataset, and their detailed description is as follows:

The set is arranged in a 2 × 2 grid of square images, each with a resolution of 250 × 250 pixels, consistent with the format in Figure 2. Like the previous set, these CT slices are taken from the axial plane, providing horizontal cross-sections of the abdomen at varying anatomical levels.

In the top left slice, portions of the kidneys are visible, with the left kidney prominently displayed. Sections of the gastrointestinal tract are also identifiable, and the spine is centrally located, providing a clear view of the vertebral body. The top right slice captures part of the liver on the left side, the spleen on the right side, and some sections of the bowel. The stomach may also be visible, depending on the level of the scan. The bottom left slice offers a clearer view of the left kidney, with the vertebra still centrally located. This slice also provides a good view of the posterior abdominal wall and adjacent structures. In the bottom right slice, both kidneys are distinctly visible, and the spine remains centrally located. The intestines and surrounding tissues are also identifiable in this view.

These images are likely contrast-enhanced, as evidenced by the bright areas, particularly in the liver, kidneys, and gastrointestinal tract, indicating high contrast uptake in these organs and their vasculature. The soft tissue structures are well differentiated due to the contrast, while bone structures, such as the vertebrae, are clearly visible. The grayscale intensities across the images show excellent contrast resolution, which allows for clear differentiation between muscle, fat, organs, and bone.

These slices provide valuable insight into the spatial relationships of major abdominal organs, with the kidneys and liver being especially prominent. This suggests that the images may be focused on assessing the function or structure of these organs. The visibility of the vertebrae in all slices also enables an assessment of the spine in the abdominal context. The images are likely used for diagnostic purposes to assess conditions such as kidney abnormalities (e.g., hydronephrosis or masses), liver conditions (e.g., hepatomegaly or lesions), or bowel issues (e.g., blockages or inflammation).

These slices provide a detailed look at key abdominal organs and would be useful for clinical diagnostics or anatomical study, given the high contrast and clear visualization of both soft tissues and bone structures.

The learning rate is a critical hyperparameter that determines how quickly the model updates its weights during training. In our proposed U-Net with residual connections, we set the initial learning rate to 0.001, with a learning rate decay strategy to ensure stable convergence. Specifically, the initial learning rate is set to 0.001 and is reduced by a factor of 0.1 every 100 epochs. The Adam optimizer is chosen due to its adaptive learning capability and its effectiveness in handling sparse gradients.

To provide a comprehensive view of the training setup, in Table 1, we list all the key hyperparameters used in the training process.

The selection of these hyperparameters is based on best practices in deep learning for biomedical image segmentation. The Adam optimizer is particularly effective as it combines the benefits of momentum and adaptive learning rates, making it well suited for training on medical image datasets. The Dice Loss + Cross-Entropy Loss combination ensures that the model optimizes shape constraints while also improving pixel-wise classification accuracy. To mitigate overfitting, a dropout rate of 0.3 is applied, which is particularly important given the relatively small dataset size. Data augmentation techniques, including rotation, flipping, cropping, and Gaussian noise, enhance the model’s ability to generalize across diverse imaging conditions and scanner variations.

Thus, we build two different models by exploiting the same U-Net architecture; the performance of each model is evaluated by using the testing dataset of the same dataset exploited for training and with the testing dataset related to the other model, resulting in four different experiments:

- Experiment I: In this experiment, we train a model with the training dataset belonging to the DI dataset and we evaluate the model trained with the testing dataset related to the DI dataset.
- Experiment II: In this experiment, we consider the model trained in the Experiment I that is evaluated with the DII dataset.
- Experiment III: In this experiment, we train a model with the training dataset belonging to the DII dataset and we evaluate the model trained with the testing dataset related to the DII dataset.
- Experiment IV: In this experiment, we consider the model trained in Experiment III that is evaluated with the DI dataset.

We consider training and testing datasets related to different datasets in experiments III and IV with the aim of showing the generalization capability of the proposed method and thus the model robustness.

For both models, we set the number of epochs equal to 500. The experiment parameters were set in an empirical way.

**Figure 4:**
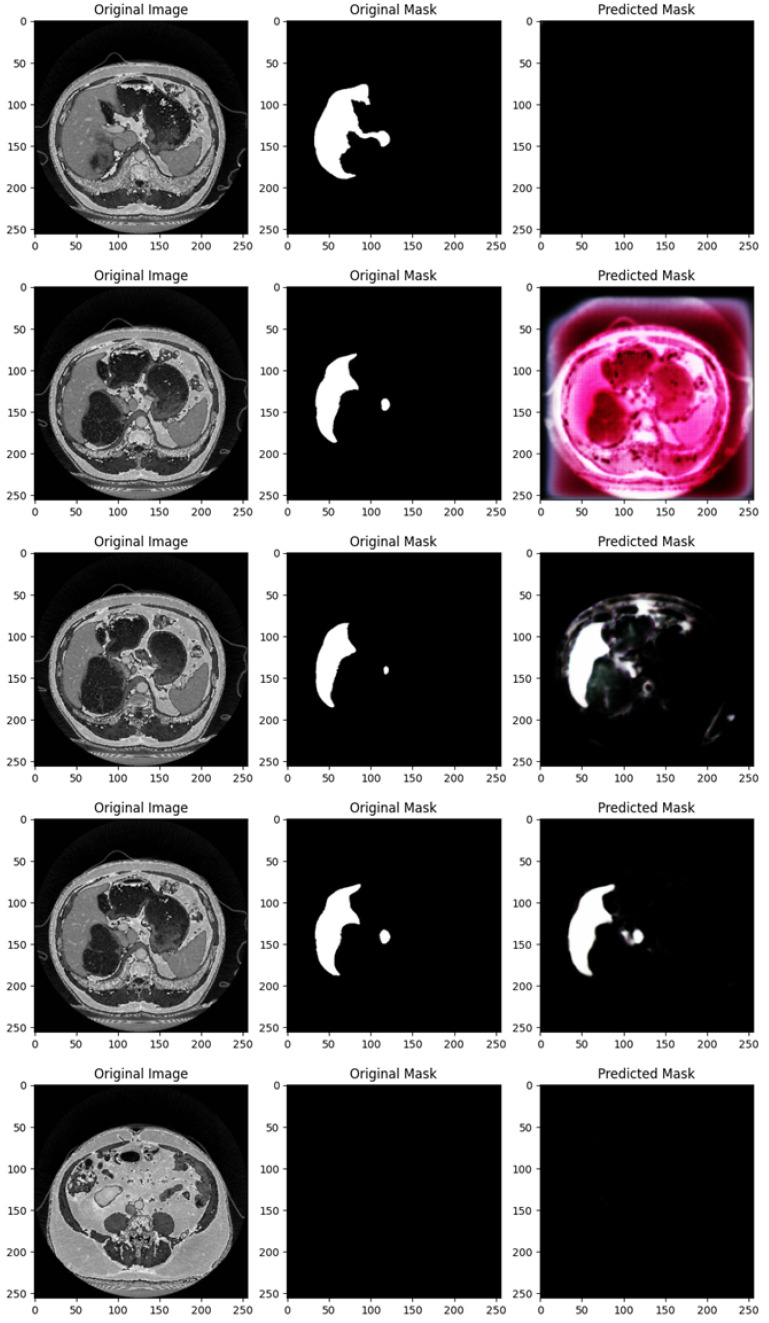
An example of segmentation mask in the U-Net trained, respectively, for 1, 25, 50, 75 and 100 epochs with the DI dataset.

Figure 4 shows the segmentation capabilities every 25 epochs (for the first 100 epochs) of the U-Net model trained with the DI dataset.

Figure 4 shows, for different epochs, a set of images with the related segmentation mask predicted at a certain epoch. In particular, it shows the original images, original masks (i.e., the ground truth) and the predicted masks generated by a U-Net network trained for different numbers of epochs (1, 25, 50, 75, and 100 from top to bottom). The aim of the U-Net network is likely to segment a specific region of interest in the abdominal CT scans, i.e., the liver, based on the shape of the masks.

In the following, we provide a detailed analysis of the several masks generated for each epoch considered in Figure 4.

At 1 epoch, the original abdominal CT scan clearly shows several internal structures. The original mask marks the liver region in white, indicating its precise location and shape. However, the predicted mask is empty, suggesting that the U-Net model has not yet learned to recognize and segment the liver region. This is expected, as early stages of training often result in poor or no predictions.

At 25 epochs, the model begins to show some improvement. The original image is another abdominal CT slice, displaying a similar view. The liver region is again marked in white in the original mask, now with a small additional structure. The predicted mask, although incomplete and somewhat noisy, shows a partial segmentation of the liver. The areas predicted by the model are highlighted in pink, indicating that while there is some correspondence with the original mask, the model is still in the process of learning to isolate the liver region correctly.

By 50 epochs, the model’s performance significantly improves. The original image is another abdominal slice, and the liver, along with a small adjacent structure, is clearly demarcated in the original mask. The predicted mask now captures a larger portion of the liver, though there is still some noise around the region, signaling that the model is not yet fully refined. However, it has made substantial progress in recognizing and outlining the liver.

At 75 epochs, the model’s segmentation is much more accurate. The original image remains consistent with the others in this set. The liver region is again highlighted with a small additional white structure in the original mask. The predicted mask now closely approximates the shape of the liver shown in the original mask, with minimal noise. The model has become much better at distinguishing the target region, though a small amount of excess segmentation remains, indicating that further refinement is possible.

At 100 epochs, a new abdominal slice is introduced. However, this slice does not have an original mask, possibly indicating a different region or a non-target slice. The predicted mask is empty, similar to the prediction after 1 epoch. Since the original mask is missing, this suggests that the model is either not designed to segment this specific structure or has successfully learned not to apply a mask where no liver structure is expected.

As shown from Figure 4, as the number of epochs increases, the U-Net network demonstrates clear improvement in segmenting the liver. At 1 epoch, the model has no predictive capability. By 25 epochs, it starts to recognize relevant areas but is still noisy. From 50 epochs onward, the predicted masks align more closely with the original masks, with improved segmentation accuracy and reduced noise. The model at 100 epochs demonstrates the ability to avoid unnecessary predictions in slices where no liver is present, indicating the model has generalized better.

Training the U-Net model over more epochs results in significant improvements in segmentation quality, with the model learning to more accurately capture the target region (liver) and minimize noise. By 75 epochs, the predictions are highly accurate, and by 100 epochs, the model shows the ability to generalize to slices where no segmentation is required, which is a sign of good model training.

**Figure 5:**
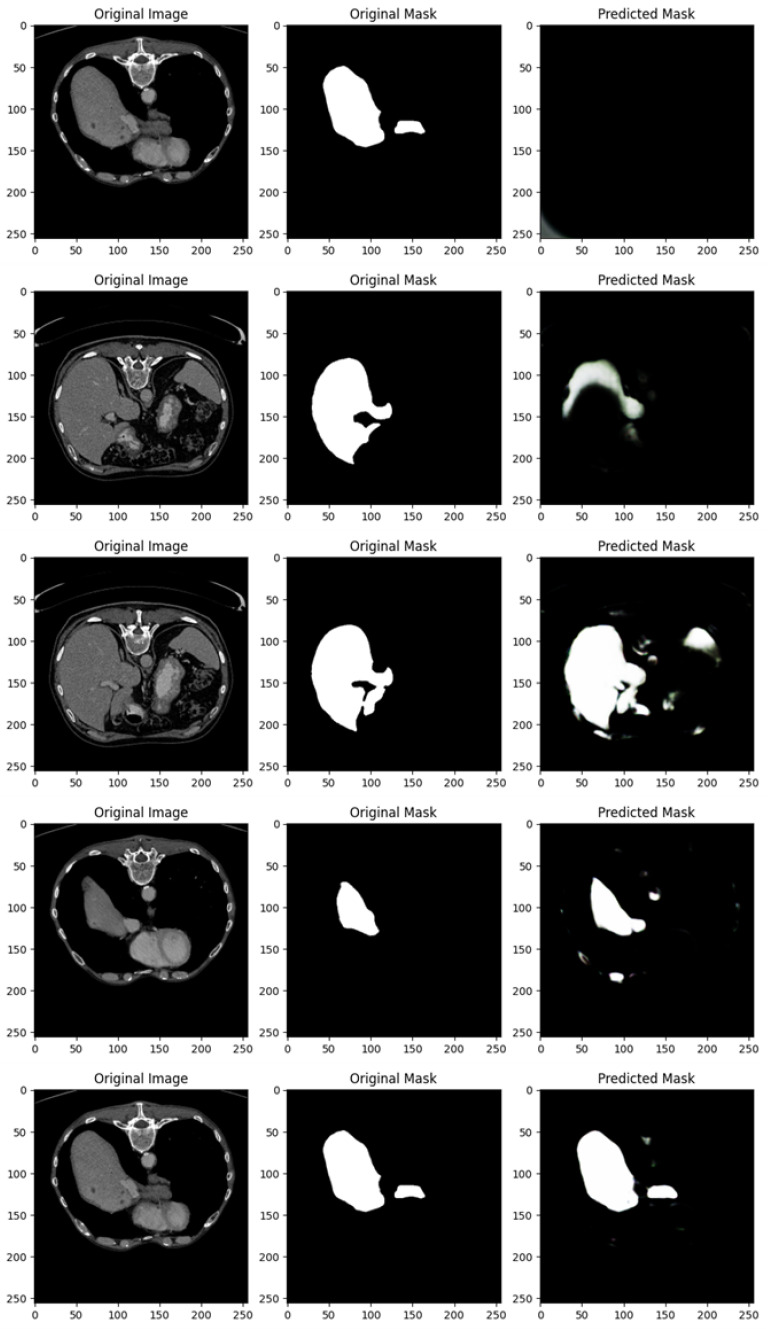
An example of segmentation mask in the U-Net trained, respectively, for 1, 25, 50, 75 and 100 epochs with the DII dataset.

Figure 5 shows the segmentation capabilities every 25 epochs (for the first 100 epochs) of the U-Net model trained with the DII dataset.

Figure 5 shows, similarly to Figure 4, for different epochs, a set of images with the related segmentation mask predicted at a certain epoch. In particular, Figure 5 shows the original images, original masks (i.e., the ground truth) and the predicted masks generated by a U-Net network trained for different numbers of epochs (1, 25, 50, 75, and 100 from top to bottom).

We provide a detailed analysis for the several masks generated for each epoch considered in Figure 5:

At 1 epoch, the CT scan shows an upper abdominal slice, and the original mask clearly marks a white region, which appears to be a segment of an organ, likely the spleen or liver. However, the predicted mask is empty, suggesting that the U-Net model, at this early stage of training, has not yet learned to recognize and predict the target region. This is typical for early-stage training models and aligns with the expected behavior of the model at this point.

At 25 epochs, the model begins to show some improvement. The original image is another abdominal CT slice with visible internal organs, and the target organ (likely the liver or spleen) is again demarcated in white. The predicted mask at this stage is partial and somewhat noisy, lacking clear boundaries. While the shape begins to resemble the organ, the prediction is still underdeveloped, which is characteristic of intermediate-stage training.

By 50 epochs, the model’s performance has significantly improved. The original image is another CT scan slice showing a similar anatomical view, and the organ of interest is clearly outlined in white. The predicted mask captures a much larger portion of the target region, and the segmentation is more refined. Despite this progress, some blurred edges and noise remain, indicating that the model is still refining its boundaries, which is expected as it continues to learn.

At 75 epochs, the model achieves a high level of accuracy. The original image is another abdominal CT slice, and the target organ is clearly marked with a smaller, localized region. The predicted mask closely resembles the original mask, with distinct boundaries and minimal noise compared to earlier stages. At this point, the U-Net model has learned to accurately isolate and segment the region of interest with greater precision.

At 100 epochs, the model demonstrates very high accuracy. The original image is an upper abdominal CT scan, and the original mask again marks the target organ with clear boundaries. The predicted mask is almost identical to the original, with sharp boundaries and very little noise or extraneous segmentation. This shows that by 100 epochs, the U-Net model is able to predict the target region with high fidelity and precision.

As shown from Figure 5, as the number of epochs increases, the U-Net network shows clear improvement in segmenting the liver organ. At 1 epoch, the model fails to make any predictions. By 25 epochs, it begins to recognize some parts of the organ, though the segmentation is noisy and incomplete. From 50 epochs onward, the segmentation becomes progressively more accurate, with the U-Net model capturing more of the organ’s shape and reducing background noise. At 75 and 100 epochs, the predicted masks closely match the original masks, showing the model’s capability to accurately segment the target region after more extensive training.

With increased training, the model becomes progressively better at recognizing and segmenting the target region. By 100 epochs, the model reaches a stage where the predicted masks are highly accurate, closely mirroring the original masks with minimal noise and strong boundary definition.

**Figure 6:**
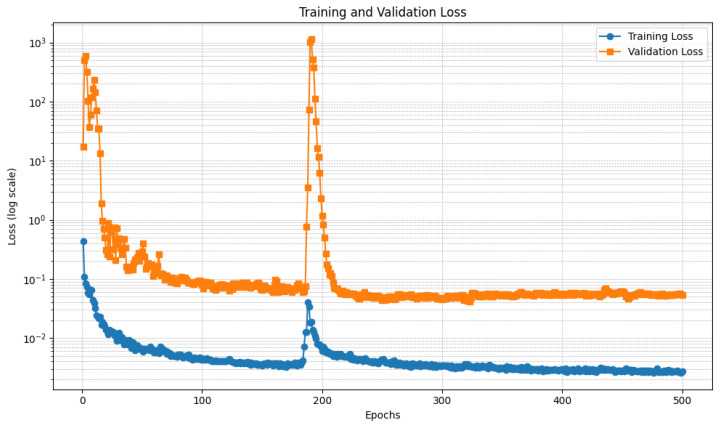
The training and the validation loss trend for the U-Net model trained with the DI dataset.

**Figure 7:**
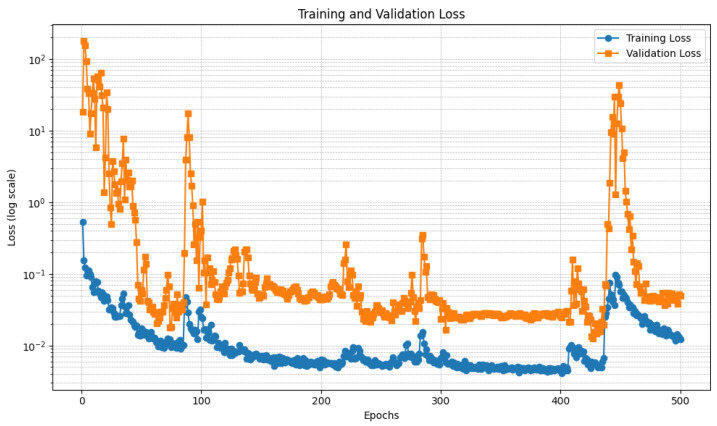
The training and the validation loss trend for the U-Net model trained with the DII dataset.

Figure 6 and Figure 7 show the trend of the loss relating to the U-Net model, respectively, trained with the DI and the DII datasets.

In detail, Figure 6 and Figure 7 present two plots showing the training loss and validation loss over 500 epochs for the U-Net models each trained on different datasets, with the y-axis in logarithmic scale to better understand the loss trend. Both plots represent how well the models are learning and generalizing during training, but they exhibit significant differences in behavior.

Regarding Figure 6, this plot depicts the training and validation loss trends over 500 epochs, with the y-axis using a logarithmic scale to accommodate the wide range of loss values. The training loss, represented by the blue curve, demonstrates a steady decrease as the training progresses, stabilizing at a low value after approximately 200 epochs. This indicates that the model is effectively learning from the training data. In contrast, the validation loss, represented by the orange curve, starts with significant fluctuations during the initial epochs. This instability is likely due to high variance in the validation data or the model’s early-stage adjustments. A notable spike is observed around epoch 200, after which the validation loss stabilizes. This spike could be linked to a sudden change in the training dynamics, such as a learning rate adjustment or a major update in the model’s parameters. By the end of the training process, both the training and validation losses converge to relatively low values. However, the final validation loss remains slightly higher than the training loss, suggesting a degree of overfitting. This gap might indicate the need for additional regularization techniques, such as dropout, early stopping, or data augmentation, to improve the model’s generalization. Overall, the plot shows that the model performs well in minimizing both training and validation loss, except for the transient instability around epoch 200. Further analysis of this spike and the training dynamics in that region could provide insights into improving model performance.

With regard to Figure 7, this plot illustrates the training and validation loss trends over 500 epochs with a logarithmic y-axis to visualize the wide range of loss values effectively. The training loss, shown in blue, demonstrates a consistent downward trend, stabilizing at a low value after approximately 100 epochs. This indicates that the model is learning effectively from the training data, with minimal fluctuations observed throughout the process. In contrast, the validation loss, represented by the orange curve, exhibits considerable variability across the epochs. During the initial phase, the validation loss starts high and fluctuates significantly, stabilizing only after approximately 100 epochs. However, there are multiple noticeable spikes throughout the training process, particularly around epochs 100, 300, and 400. These spikes suggest disruptions in training dynamics, possibly due to changes in the learning rate, issues with batch selection, or sensitivity to certain patterns in the validation set. Despite the fluctuations, the overall trend shows that the validation loss stabilizes to relatively low values in later epochs, indicating reasonable generalization. However, the final validation loss remains consistently higher than the training loss, which could point to slight overfitting. Addressing this may involve incorporating regularization methods, adjusting the learning rate schedule, or increasing the validation data’s representativeness. Overall, while the model shows effective learning and generalization trends, the recurring spikes in validation loss require further investigation. Identifying the root cause of these spikes could help improve model stability and overall performance.

Relating to the differences between the two U-Net models, the first plot indicates more stable validation performance, with only a single notable spike in the validation loss before stabilization. This suggests that the training process was smoother and potentially better tuned, possibly due to a more effective learning rate schedule, data preparation, or model architecture. The second plot, however, shows recurring validation loss spikes, indicating that the model experienced more difficulty in maintaining stable performance across the validation set. These fluctuations could result from an unstable training process, sensitivity to specific validation batches, or an inadequately tuned learning rate. Despite the differences, both plots demonstrate effective learning, as evidenced by the consistent decrease in training loss and the eventual convergence of validation loss. However, the second plot’s instability suggests the need for further investigation into the training dynamics. Strategies such as refining the learning rate schedule, increasing the validation set’s size or diversity, or employing techniques like early stopping might help mitigate the observed fluctuations and improve overall stability.

We analyze each experiment into a distinct subparagraph, where we discuss the results and we provide a visual impact related to the mask prediction performed by the proposed models.

### 4.5. Experiment I Results

In this section, we provide the experimental analysis results related to Experiment I. The testing accuracy in this experiment is equal to 0.8129, the Dice coefficient is equal to 0.788 and the IoU is equal to 0.65.

**Figure 8:**
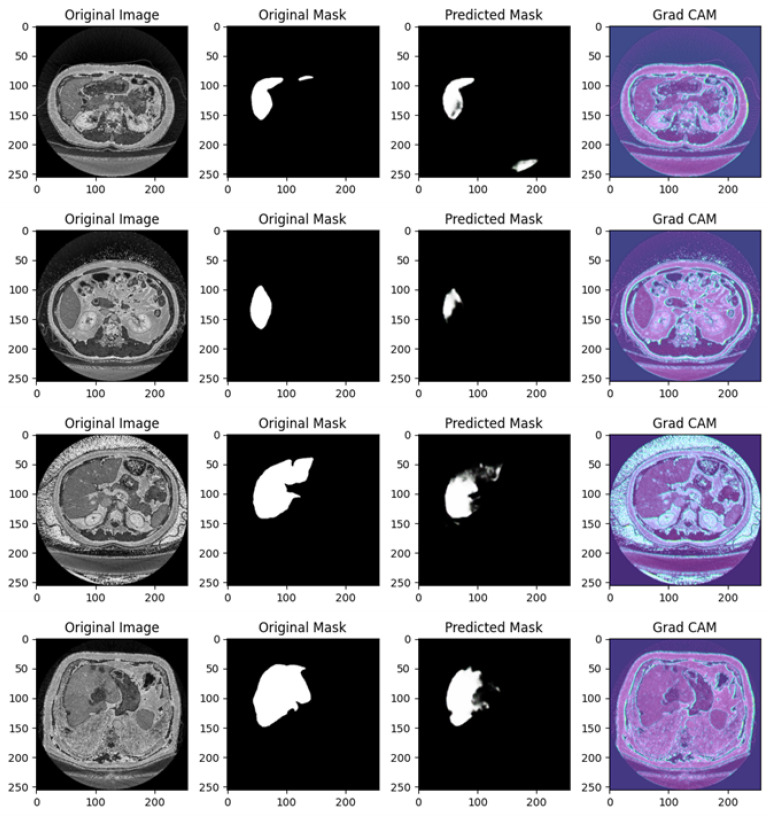
Examples of prediction related to Experiment I, i.e., with images obtained with the model trained on images belonging to the DI dataset with testing images related to the DI dataset.

Figure 8 shows several examples of prediction related to Experiment I.

Figure 8 shows different examples of segmentation performed by the proposed method. In particular, it is related to four different cases, each including the original image, original mask, predicted mask, and a Grad-CAM visualization.

In the following, we provide a detailed analysis for each example of prediction shown in Figure 8:

Example of Prediction 1: The CT scan shows an abdominal cross-section, and the original mask marks a small, distinct organ or lesion in white. The U-Net model’s prediction shows partial success; however, the predicted region is smaller and incomplete compared to the original mask, with some background noise visible in the lower right corner. The Grad-CAM heatmap highlights areas the model deems important for making its prediction. The regions in blue and purple have higher activation and focus primarily around the central abdominal organs. However, the important areas do not align perfectly with the true region of interest, suggesting that the model is not fully capturing the relevant structures.

Example of Prediction 2: In a different CT scan showing another cross-sectional view of the abdomen, the original mask isolates a well-defined small structure, likely an organ, in white. The model’s prediction in this case is less accurate than in the previous example, with a significant under-prediction. Only a faint, noisy portion of the original mask is captured. The Grad-CAM heatmap indicates that the model is activating over certain areas but appears to miss the actual region of interest. The model’s focus on other parts of the image likely contributes to the poor segmentation in this instance.

Example of Prediction 3: This CT scan shows a broader abdominal region with more complex anatomy. The target organ or lesion is segmented much more prominently and clearly in the original mask. The U-Net model performs better here, capturing a significant portion of the organ or lesion. However, the prediction is still slightly smaller than the original mask, with some blurred edges and missing parts. The Grad-CAM activation map is well aligned with the central portion of the image, focusing on regions relevant to the segmentation task. This explains the improvement in prediction accuracy compared to earlier examples, as the model is now focusing on more pertinent areas of the image.

Example of Prediction 4: In another cross-sectional CT scan of the abdomen, the original mask highlights a medium-sized organ or lesion segmented in white. The prediction is quite accurate, capturing the main structure well, but still slightly under-segmented compared to the original mask. There are minor discrepancies, with some noise around the edges. The Grad-CAM heatmap shows a good focus on the relevant areas, with the model attending to regions near the original mask, which explains the more precise segmentation in this case.

The U-Net model demonstrates a mixed ability to accurately predict the regions of interest. In some cases (like in examples of predictions 3 and 4), the predictions are relatively close to the original mask, but in others (such as examples of predictions 1 and 2), there are clear deficiencies, with significant under-segmentation and background noise. Grad-CAM provides a valuable insight into the decision-making process of the model. In examples of prediction where the segmentation is more accurate (such as examples of predictions 3 and 4), the Grad-CAM heatmaps show a stronger focus on the relevant regions, while in poorer predictions (examples of predictions 1 and 2), the activations are misaligned with the actual target region, which contributes to incorrect segmentation. The quality of predictions improves as the Grad-CAM focus becomes more aligned with the region of interest.

### 4.6. Experiment II Results

In this section, we provide the experimental analysis results related to Experiment II. The testing accuracy in this experiment is equal to 0.8876, with a Dice coefficient equal to 0.857 and with an IoU equal to 0.75.

**Figure 9:**
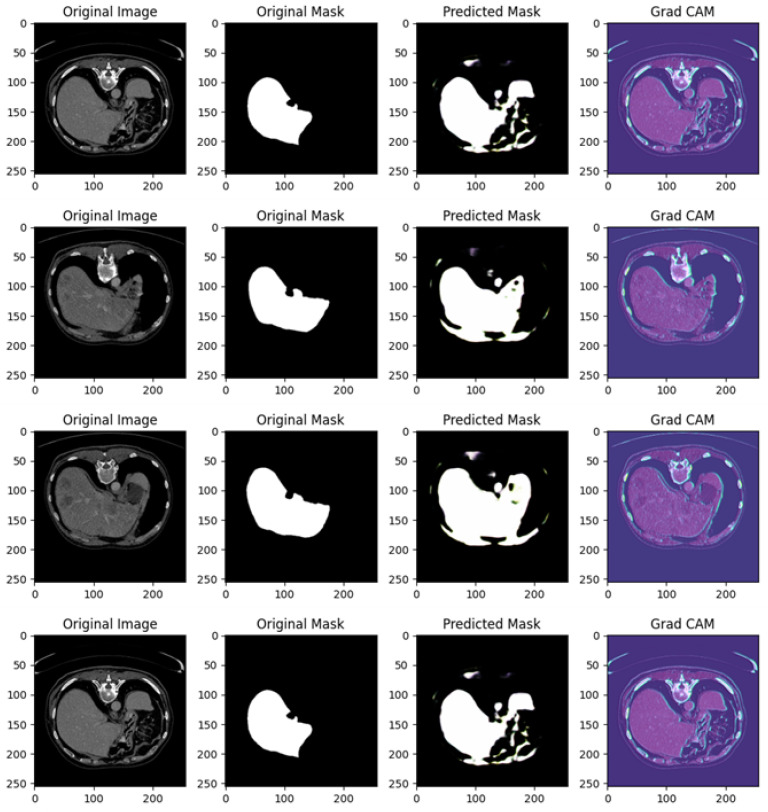
Examples of prediction related to Experiment II, i.e., with images obtained with the model trained on images belonging to the DI dataset with testing images related to the DII dataset.

Figure 9 shows several examples of prediction related to Experiment II.

Figure 9 in related to a comprehensive analysis of the segmentation predictions of a U-Net model across four different cases, each including the original image, original mask, predicted mask, and a Grad-CAM visualization.

In the following, we provide a detailed analysis for each example of prediction shown in Figure 9; this analysis showcases how well the U-Net network is performing in identifying and segmenting regions of interest in abdominal CT scans, as well as the model’s focus during predictions.

Example of Prediction 1: The CT scan shows a cross-sectional view of the abdomen, with the target area, likely a liver or lesion, well-defined and highlighted in white in the original mask. The U-Net model captures a significant portion of the target region, but over-segmentation artifacts are present, with additional highlighted areas not found in the original mask. The prediction is somewhat noisy, and parts of the background are incorrectly included in the segmentation. The Grad-CAM heatmap reveals high activations around the central liver area, indicating that the model is focusing on the correct region. However, significant activation in peripheral areas may explain the incorrect predictions in the background.

Example of Prediction 2: In a different abdominal CT scan, showing a similar anatomical structure to the first example, the original mask clearly segments the organ of interest, likely the liver. The U-Net prediction closely resembles the original mask, though small regions around the edges show slight over-segmentation, and there is some minor noise. The model appears to correctly identify the boundaries of the target region, although not perfectly. The Grad-CAM heatmap highlights a strong focus on the liver area, with concentrated activations near the region of interest, reflecting the improved segmentation accuracy compared to the previous example.

Example of Prediction 3: This image shows a cross-sectional view of the abdomen with greater contrast than the previous scans. The original mask outlines a large portion of the target organ accurately. The prediction is highly accurate, with minimal over-segmentation or noise present. The U-Net model performs well, capturing the organ’s structure almost perfectly, with slight discrepancies only at the edges. The Grad-CAM heatmap indicates a precise focus on the relevant areas, with the model’s attention well concentrated on the organ of interest, which explains the high prediction accuracy. Peripheral activations are minimal, leading to reduced noise.

Example of Prediction 4: Another similar cross-sectional abdominal scan is presented, with the original mask clearly segmenting the target organ. The predicted mask captures the main structure, but, as in the first example, over-segmentation artifacts are present. The model has overextended the segmentation, including areas outside the original mask, although the general shape of the organ is correctly identified. The Grad-CAM heatmap shows a strong focus on the central region, but some activations are spread across the periphery, particularly in regions not part of the target area. This suggests that the over-segmentation is likely due to the model’s attention being scattered across the image.

The U-Net model is generally successful at predicting the organ of interest but tends to over-segment in certain instances (as seen in prediction examples 1 and 4). This could be due to background features or noise influencing the model’s predictions. The Grad-CAM visualizations are instrumental in identifying where the model’s attention is focused during the segmentation process. In examples showing better predictions (i.e., 2 and 3), the heatmap shows concentrated activations on the target regions. In predictions with more over-segmentation (i.e., 1 and 4), the model’s focus is more dispersed, leading to less precise predictions.

The quality of the predictions varies, with examples of predictions 2 and 3 showing near-perfect segmentations, while examples of predictions 1 and 4 display more noise and over-segmentation. The Grad-CAM maps corroborate this by indicating that the model’s focus aligns better with the target areas in examples of predictions 2 and 3.

The U-Net model’s segmentation performance shows strong potential but is somewhat affected by over-segmentation in some cases. The Grad-CAM visualizations offer valuable insights into the model’s decision-making process, indicating that further refinement could be made to improve the focus on the target regions and reduce noise in the predictions. Overall, the model demonstrates good performance, especially in examples of predictions 2 and 3.

### 4.7. Experiment III Results

In this section, we provide the experimental analysis results related to Experiment III. The testing accuracy in this experiment is equal to 0.8948, with a Dice coefficient equal to 0.888 and an IoU metric with a value of 0.80.

**Figure 10:**
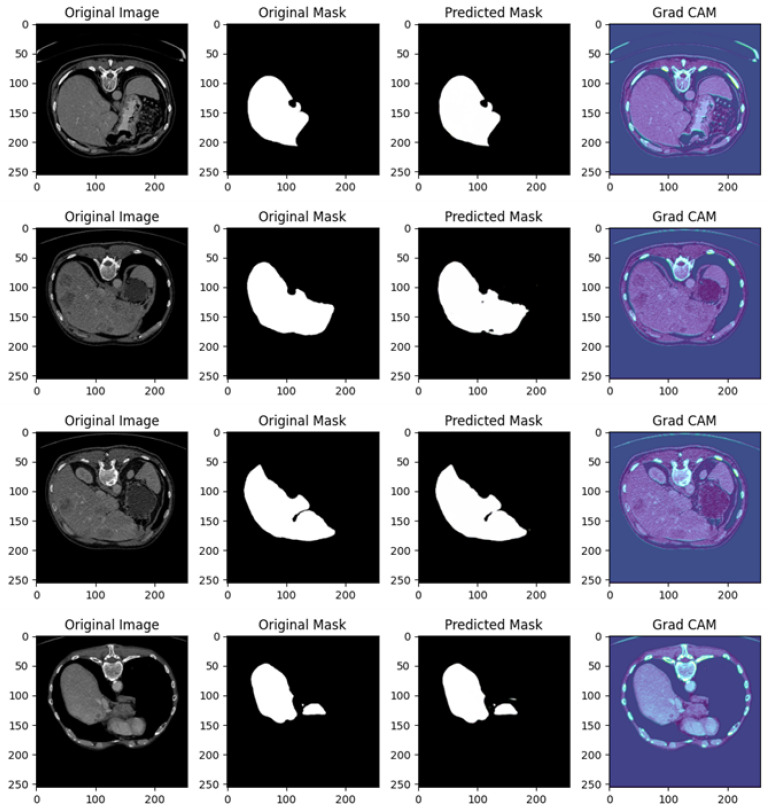
Examples of prediction related to Experiment III, i.e., with images obtained with the model trained on images belonging to the DII dataset with testing images related to the DII dataset.

Figure 10 shows several examples of prediction related to Experiment III.

Figure 10 is related to a comprehensive analysis of the segmentation predictions of a U-Net model across four different cases, each including the original image, original mask, predicted mask, and a Grad-CAM visualization.

Example of Prediction 1: The CT scan shows a cross-sectional view of the liver region, with surrounding anatomy clearly visible. In the original mask, the target organ (the liver) is accurately segmented. The U-Net model’s prediction closely follows the shape of the original mask, but there is a small under-segmentation issue, particularly around the edges of the liver. While the shape and size are mostly consistent, some fine details are missing. The Grad-CAM heatmap reveals that the model predominantly focuses on the liver region. However, there are some peripheral areas of activation outside the liver, which may explain the minor segmentation inaccuracies.

Example of Prediction 2: Another CT scan provides a slightly different perspective of the abdomen. The original mask segments the target organ over a larger area compared to the first row. The U-Net model’s prediction is very close to the original mask, capturing almost the entire organ accurately. Though there is a slight discrepancy in the boundary, the prediction is overall more accurate than the first one. The Grad-CAM heatmap shows that the model’s attention is well concentrated on the target organ, with minimal distractions outside the region of interest. This focused attention explains the good quality of the prediction.

Example of Prediction 3: This CT scan shows another cross-sectional view of the abdomen, where the original mask highlights a larger organ with precise contours. The U-Net model’s prediction performs well, capturing most of the organ. However, there is minor over-segmentation, with the model including some extraneous areas that do not belong to the target organ. Despite this, the overall shape of the organ is well preserved. The Grad-CAM heatmap shows strong activations around the liver area, but there are also peripheral regions activated, which correlates with the over-segmentation observed in the prediction.

Example of Prediction 4: This scan presents a slightly altered view of the abdomen, similar to the previous rows. In the original mask, the liver is clearly outlined. The U-Net prediction shows under-segmentation, where the model fails to capture the entire organ, especially around the edges. The size of the predicted mask is notably smaller than the original mask. The Grad-CAM heatmap indicates that the model’s focus is somewhat diffused, with significant attention placed on regions outside the target organ, which likely explains the under-segmentation seen in the predicted mask.

The U-Net model performs well overall, but the predictions show some variability. Prediction examples 2 and 3 display better segmentation results, with only minor deviations in boundary precision, while prediction examples 1 and 4 exhibit under-segmentation issues, where parts of the organ are missed.

In prediction examples 1 and 4, there is under-segmentation, meaning the model fails to capture the full extent of the target organ. In prediction example 3, over-segmentation is observed, where parts outside the target area are mistakenly included.

The Grad-CAM visualizations provide valuable insights into the model’s decision-making process. Prediction examples 2 and 3 show that the model focuses well on the relevant regions, leading to more accurate predictions. However, in prediction examples 1 and 4, the Grad-CAM maps reveal that the model is focusing on regions beyond the target, resulting in less precise predictions.

The U-Net model exhibits promising segmentation performance across the images, particularly in prediction examples 2 and 3, where the predictions closely match the original masks. The Grad-CAM visualizations effectively highlight areas where the model’s attention is misdirected, offering useful guidance for model tuning and enhancement.

### 4.8. Experiment IV Results

In this section, we provide the experimental analysis results related to the Experiment IV. The testing accuracy in this experiment is equal to 0.9305, with a Dice coefficient equal to 0.85 and an IoU of 0.85.

**Figure 11:**
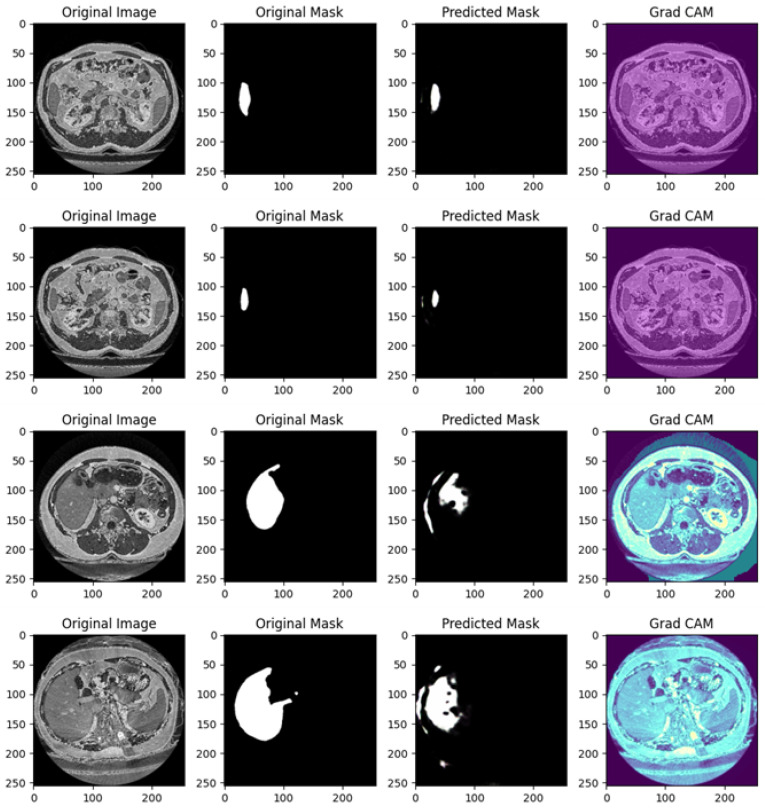
Examples of prediction related to Experiment IV, i.e., with images obtained with the model trained on images belonging to the DII dataset with testing images related to the DI dataset.

Figure 11 shows several examples of prediction related to Experiment IV.

Figure 11 is related to a comprehensive analysis of the segmentation predictions of a U-Net model across four different cases, each including the original image, original mask, predicted mask, and a Grad-CAM visualization.

Example of Prediction 1: The CT scan shows an image of the abdomen with the liver as the region of interest. The original mask displays a very small target area that corresponds to a specific structure. However, the U-Net model’s prediction only partially captures the structure, showing under-segmentation. It does not fully cover the target area, and the shape of the predicted mask does not perfectly match the original. The Grad-CAM heatmap reveals that the model’s attention is somewhat diffuse, focusing on the target region but extending to a broader area. This diffuse focus may explain why the predicted mask is incomplete, as the attention is not entirely centered on the target structure.

Example of Prediction 2: Another CT scan provides a different cross-section of the abdomen. The original mask is less extensive than in the first example, but it clearly outlines the organ structure. The U-Net model’s prediction shows some under-segmentation, although it captures more of the structure compared to the first row. Despite this, the boundaries are not well defined, and the predicted region is smaller than the original mask. The Grad-CAM heatmap indicates that the model’s attention is focused on the organ of interest, similar to previous heatmaps, with some peripheral regions included. This may explain why the model struggles with precise boundary detection in its predictions.

Example of Prediction 3: This CT scan offers a slightly different view of the abdomen. The original mask segments the organ with accurate contours. The predicted mask is more accurate than in previous rows but still exhibits over-segmentation. The prediction includes areas outside the target structure, suggesting that the model has captured additional regions that were not part of the original mask. The Grad-CAM heatmap shows that the model is strongly focused on the target region, but some activation extends beyond the intended structure. This extra activation corresponds to the over-segmentation observed in the predicted mask.

Example of Prediction 4: Another CT scan presents a different cross-section of the abdomen. The original mask is well defined, covering a larger region compared to earlier examples. However, the U-Net model’s prediction exhibits significant over-segmentation. The prediction exceeds the boundaries of the original mask and includes non-target areas. The Grad-CAM heatmap shows that the model is focusing not only on the organ of interest but also on the surrounding areas. This broader focus likely contributes to the over-segmentation observed in the predicted mask.

The U-Net model exhibits mixed performance. In the first two prediction examples, the model struggles with under-segmentation, where it fails to capture the entire organ, leading to incomplete masks. In the third and fourth rows, the model suffers from over-segmentation, where it captures more than just the target area, predicting larger masks than needed. The segmentation inaccuracies vary between under-segmentation (first and second prediction examples) and over-segmentation (third and fourth prediction examples). This suggests the model struggles with precise boundary detection, either missing parts of the target or including too much of the surrounding area.

The Grad-CAM visualizations offer insight into the model’s focus areas. In the first two prediction examples, the model’s attention is dispersed, contributing to the under-segmentation observed. In the last two prediction examples, the model’s attention is broader, extending beyond the intended target, which may explain the over-segmentation. These heatmaps are useful for diagnosing where the model’s attention drifts outside the relevant area.

The U-Net model demonstrates varying degrees of segmentation accuracy in this set of images. It struggles with under-segmentation in the first two rows, likely due to diffused attention seen in the Grad-CAM heatmaps. In the third and fourth rows, the model exhibits over-segmentation, with Grad-CAM visualizations showing excess attention outside the target organ. Refining the model to better focus its attention within the boundaries of the target structure could lead to improved segmentation performance and reduce both under- and over-segmentation issues.

The proposed method demonstrates promising capabilities, but certain areas warrant further analysis and improvement. One interesting challenge in the experimental analysis relates to the potential for overfitting during the training process, which can undermine the robustness and generalizability of the model. The experimental results highlight specific observations about the behavior of the model and its robustness across datasets. The generalization capabilities of the approach are interesting, with accuracies ranging between 0.81 and 0.93 across experiments. This demonstrates its potential for handling variability in imaging conditions also acquired with different medical equipment. However, the validation loss spike observed during training raises concerns about stability. This spike suggests that the model may temporarily overfit to patterns in the training data, and although it recovers, the instability warrants a more detailed analysis. Adjusting the learning rate schedule or incorporating more robust early stopping criteria might address this issue.

Explainability is a critical aspect of the proposed method, as demonstrated through the Grad-CAM visualizations. These heatmaps reveal that the model focuses on anatomically relevant areas, enhancing trust in its predictions. However, in some instances, Grad-CAM outputs indicate that the model’s attention extends to irrelevant regions, leading to over-segmentation.

The clinical relevance of the proposed approach is underscored by its ability to integrate into workflows for preoperative planning, disease progression monitoring, and therapy assessment. With high accuracy and explainability, the model has significant potential to assist clinicians in making informed decisions.

To evaluate the performance of the proposed liver segmentation method, several key metrics were considered:

- Dice Coefficient (DSC): The Dice coefficient values range from 0.788 to 0.92, depending on the experimental setup.
- Intersection over Union (IoU)/Jaccard Index: The reported IoU values vary between 0.65 and 0.88, representing the degree of overlap between the predicted and ground truth segmentations.

These metrics provide a comprehensive evaluation of segmentation accuracy, overlap, and boundary precision, ensuring robust performance analysis.

### 4.9. Explainability Analysis

The adoption of Grad-CAM enhances the prediction explainability of the proposed liver segmentation method by highlighting regions within the input images that significantly influence the predictions of the proposed model.

The integration of Grad-CAM with the U-Net model provides an explainability layer to the proposed liver segmentation method. Grad-CAM highlights regions in the input images that significantly influence the segmentation decisions, offering a dual perspective: an understanding of the model’s internal decision-making process and insights into its clinical implications.

From the model point of view, the Grad-CAM outputs demonstrate areas of focus and reveal instances of both alignment and misalignment between the model’s attention and the ground truth segmentation. For example, in Figure 8, Grad-CAM outputs for predictions from Experiment I highlight that the model’s attention is often concentrated on the central liver region during successful segmentations. However, the heatmaps also reveal peripheral activations in some cases, such as Example 1, which correspond to over-segmentation in the predicted masks. In Example 3, the Grad-CAM activations align well with the target liver region, resulting in a highly accurate prediction. These instances underscore the importance of targeted attention and reveal opportunities for refining the model to improve its focus.

Similarly, in Figure 9, which pertains to Experiment II, Grad-CAM visualizations demonstrate improved model focus. Examples 2 and 3 show that the heatmaps closely correspond to the actual liver boundaries, resulting in accurate segmentations. However, Example 4 reveals scattered activations in peripheral areas, correlating with over-segmentation. These observations suggest that while the model’s generalization capability across datasets is promising, occasional misdirected attention can lead to inaccuracies.

From the clinician’s perspective, Grad-CAM enhances trust in the model’s predictions by providing visual evidence of the decision-making process. Accurate heatmaps, such as those in Figure 10 (Experiment III, Examples 2 and 3), reassure clinicians that the model bases its segmentation on biologically plausible and anatomically relevant regions. In contrast, the under-segmentation seen in Example 4 is accompanied by Grad-CAM heatmaps showing reduced focus on the liver region and misplaced attention on surrounding structures. This information is crucial for clinicians to identify cases requiring manual review, ensuring that the automated segmentation aligns with clinical standards.

Figure 11 from Experiment IV provides further insights into the model’s robustness. Grad-CAM visualizations in Examples 2 and 3 indicate strong attention on the liver, corresponding to precise segmentations. However, Example 1 highlights diffuse activations beyond the liver’s boundaries, which explain the under-segmentation observed in the predicted mask. These examples illustrate that while the model performs well overall, certain cases require refinement to enhance its reliability.

Specific examples in the study illustrate these dynamics:

In instances of under-segmentation, such as in Figure 8 (Experiment I, Example 1) and Figure 11 (Experiment IV, Example 1), Grad-CAM maps reveal reduced activation within the liver region and misplaced focus on surrounding structures. This indicates the model’s struggle to fully capture the target area, which might stem from insufficient training data or a lack of robust feature representation for specific anatomical variations.

Cases of over-segmentation, such as in Figure 9 (Experiment II, Example 4) and Figure 10 (Experiment III, Example 3), correspond to Grad-CAM outputs showing diffuse activations beyond the liver’s anatomical boundaries. This behavior suggests that the model is influenced by non-relevant features, which could be mitigated by incorporating stricter regularization techniques or refining the loss function to penalize erroneous activations.

In summary, the explainability offered by Grad-CAM is not only a valuable diagnostic tool for evaluating model performance but also an essential bridge for fostering trust and collaboration between AI systems and medical professionals. By pinpointing the exact regions influencing segmentation, Grad-CAM ensures that the proposed method is not only accurate but also interpretable and clinically viable.

## 5. Discussion

Liver segmentation has been a critical area of focus in medical imaging research due to its relevance in diagnosing and treating hepatic diseases. Traditional methods relied heavily on manual segmentation performed by radiologists, which is time-consuming and prone to inter-observer variability. To address these challenges, various computational approaches have been developed over the years.

Initial automated segmentation techniques used rule-based algorithms and statistical shape models, such as active contour models and level sets. While these approaches showed promise, they struggled with complex anatomical variations and noise in medical images, leading to suboptimal segmentation accuracy. The advent of machine learning introduced supervised learning algorithms, including support vector machines (SVMs) and random forests, which improved segmentation performance but required extensive feature engineering.

With the rise of deep learning, convolutional neural networks (CNNs) have become the dominant paradigm for medical image segmentation. The U-Net architecture [30] revolutionized biomedical image segmentation by employing an encoder–decoder structure with skip connections to retain spatial information. U-Net and its variants, such as 3D U-Net and attention U-Net, have been widely adopted for liver segmentation. For instance, Christ et al. [31] developed a cascaded U-Net for liver and tumor segmentation.

Attention mechanisms have further enhanced segmentation performance by enabling models to focus on relevant regions within medical images. Works by Oktay et al. [32] integrated attention gates into the U-Net architecture to refine segmentation outputs, particularly for small and complex structures. Additionally, hybrid approaches combining CNNs with transformer-based architectures have recently emerged, demonstrating superior performance in capturing global context.

Liver segmentation from medical images, such as computed tomography (CT) and magnetic resonance imaging (MRI), plays a crucial role in diagnosis and treatment planning for liver diseases, including liver tumors and cirrhosis. Recent advancements in deep learning have shown significant promise in improving the accuracy and efficiency of liver segmentation, with methods often based on convolutional neural networks (CNNs) and variants such as UNet, VNet, and attention-based mechanisms.

In the context of medical image segmentation, the U-Net model [30] is widely recognized for its effectiveness in medical image segmentation, owing to its encoder–decoder structure that captures both local and global features. Over the years, various enhancements and adaptations of the U-Net model have been proposed to tackle the specific challenges of liver segmentation, such as shape variability, texture heterogeneity, and low contrast in CT images.

The original U-Net [30] laid the foundation for numerous subsequent works in liver segmentation. Following this, several modifications were introduced to improve performance in challenging conditions. For example, Milletari et al. [33] proposed the V-Net, which extended U-Net to 3D segmentation by using volumetric convolutions and a Dice coefficient-based loss function. This approach demonstrated superior performance in 3D liver segmentation tasks.

Authors in Kavur et al. [34] conducted an extensive comparison of liver segmentation algorithms on multiple datasets, highlighting the strengths and limitations of each method. Their study showed that while 2D CNN-based methods, like traditional UNet, are efficient and computationally less expensive, 3D CNNs and hybrid approaches are better suited for capturing volumetric details essential for accurate liver segmentation.

Table 2 directly compares the state-of-the-art research works in the context of liver segmentation.

As shown from the state-of-the-art comparison shown in Table 2, the proposed method is able to obtain interesting performances.

In medical applications, the explainability of deep learning models is paramount for gaining clinician trust and improving the interpretability of predictions. Gradient-weighted Class Activation Mapping (Grad-CAM) [25] is one of the most widely used techniques for visualizing the regions of an image that contribute most to the model’s decisions. While commonly applied in classification tasks, its integration into medical image segmentation models is an emerging research area.

As a matter of fact, there has been limited exploration of explainability in liver segmentation tasks. The proposed method addresses this gap by incorporating Grad-CAM with a U-Net architecture specifically tailored for liver segmentation, providing visual explanations that can aid clinicians in understanding and verifying the segmentation results.

The choice of U-Net with residual connections in this study is well justified, as it offers several advantages for medical image segmentation, particularly in liver CT scans. However, we acknowledge that additional architectural variations, such as attention mechanisms and transformer-based segmentation models, could further enhance performance. Below, we provide a justification for our chosen architecture while discussing potential improvements.

One of the key advantages of U-Net with residual connections is its ability to preserve fine-grained anatomical details. The encoder–decoder structure of U-Net effectively captures both global and local image features, which is particularly beneficial for segmenting the liver, an organ with complex boundaries. Moreover, the incorporation of residual connections improves gradient flow, addressing the issue of vanishing gradients and ensuring that the network learns fine details without losing spatial information.

Another important consideration is the efficiency of training on limited data. Medical datasets are often small, making deep networks susceptible to overfitting. Compared to transformer-based models, U-Net maintains a moderate level of complexity, allowing it to achieve high accuracy even with fewer training samples. The presence of skip connections further enhances the model’s performance by retaining low-level spatial features from the encoder and reintroducing them in the decoder, ultimately improving segmentation accuracy despite the limited dataset size.

In addition to its accuracy, U-Net with residual connections offers computational efficiency, a crucial factor for clinical applications. While transformer-based models, such as Swin-UNet, have demonstrated state-of-the-art segmentation results, they require significantly higher computational resources, which may not always be available in real-world medical settings. By contrast, U-Net with residual connections strikes a balance between accuracy and efficiency, making it a practical choice for medical imaging tasks where computational constraints are a concern.

The U-Net model with residual connections and Grad-CAM explainability, employed in this study, is specifically designed for liver segmentation. However, its outputs can be seamlessly integrated with other AI-based diagnostic models, including those for lesion classification and survival prediction. The ability to segment liver structures with high precision enhances the performance of these models by providing more reliable region-of-interest data and improving interpretability.

Lesion classification models typically depend on either global image features or manually defined regions of interest. The proposed segmentation method automates the extraction of these regions, ensuring that lesion classification models focus solely on liver areas rather than extraneous background structures. This automation reduces the likelihood of false positives and increases classification accuracy, particularly in cases involving complex liver textures or overlapping anatomical structures. Deep learning models such as CNN-based classifiers, including ResNet, VGG, and EfficientNet, can leverage the segmented liver regions to categorize lesions as benign, malignant, or cystic. Transformer-based models, such as Vision Transformers and Swin Transformers, can further enhance classification performance by capturing high-level spatial dependencies within segmented liver regions. Hybrid CNN-RNN models, designed to analyze time-series data, can utilize follow-up scans to track lesion progression or regression. The proposed segmentation approach benefits these classification models by automating lesion localization, improving feature extraction by reducing background noise, and providing explainable segmentation masks that clinicians can verify before proceeding with classification.

Beyond lesion classification, segmentation also plays a crucial role in survival prediction models. These prognostic models typically analyze patient-specific factors such as tumor size, liver volume, lesion count, and radiomic features to estimate survival probabilities. The segmentation outputs contribute to this analysis by extracting quantitative imaging biomarkers, including tumor volume, liver-to-tumor ratio, texture-based radiomic features that capture heterogeneity and intensity distribution, and shape descriptors reflecting tumor boundary irregularities. Machine learning techniques such as Random Forests, Support Vector Machines, and XGBoost can process segmentation-derived features to predict survival time. Meanwhile, deep learning models, including recurrent neural networks and long short-term memory networks, can track tumor growth patterns over multiple time points. Multimodal AI approaches that integrate convolutional neural networks with patient demographics, laboratory results, and genetic data offer a more comprehensive survival analysis. The integration of segmentation data into these models ensures precise tumor and liver delineation, improves interpretability by highlighting regions influencing survival risk, and supports longitudinal tracking of disease progression for personalized prognosis.

Despite these advantages, several challenges must be addressed to optimize integration with lesion classification and survival prediction models. Data standardization remains a significant concern, as variations in imaging protocols across different datasets require harmonization before integration. Additionally, survival prediction models may need segmentation beyond the liver, particularly for detecting vascular invasion or distant metastases. Another challenge is the computational complexity introduced by combining segmentation with classification or survival analysis models. Optimization techniques such as model pruning and the development of lightweight architectures could help mitigate these computational demands.

Future advancements in this area could focus on enhancing model fusion by developing end-to-end pipelines that integrate liver segmentation with lesion classification models. The exploration of multimodal AI approaches that combine imaging data with omics and clinical biomarkers could further refine survival prediction. Expanding the segmentation model to multi-phase CT and MRI data could provide more detailed liver lesion characterization and enable dynamic tumor assessment.

Overall, the proposed explainable U-Net segmentation model establishes a strong foundation for integration with lesion classification and survival prediction systems. By automating liver region extraction and improving interpretability, it enhances AI-driven diagnostic workflows, leading to more precise, reliable, and clinically valuable predictions.

In the following, we compare the proposed method with state-of-the-art deep learning models and highlight its advantages over recent developments.

U-Net is widely used for biomedical segmentation due to its encoder–decoder architecture with skip connections, making it effective even when trained on limited datasets. However, it struggles with fine-grained details and complex boundaries, leading to suboptimal segmentation in cases involving tumors, lesions, or low-contrast regions. Furthermore, its lack of interpretability makes it difficult for clinicians to trust AI-generated segmentation results.

Enhancement in Our Approach: The integration of residual connections improves feature propagation and prevents vanishing gradients, resulting in better boundary preservation. Additionally, the use of Grad-CAM enhances explainability, allowing medical professionals to visualize which regions influence the segmentation decision.

Attention U-Net introduces attention gates to focus on relevant liver regions, reducing false positives. However, it is computationally more expensive, making real-time clinical deployment challenging, and still operates as a black box without explicit interpretability.

Enhancement in Our Approach: While attention mechanisms improve feature selection, our approach maintains computational efficiency by using residual connections instead of attention gates. Furthermore, Grad-CAM provides post hoc interpretability, which is absent in Attention U-Net.

Transformer-based models, such as TransUNet and Swin-UNet, leverage self-attention mechanisms to capture global context, making them highly effective for segmenting complex structures. They are also more robust to variations in imaging conditions. However, they require large datasets and extensive computational resources, limiting their usability in clinical settings. Furthermore, they remain less interpretable compared to CNN-based models.

Enhancement in Our Approach: While transformers excel in capturing long-range dependencies, our U-Net with residual connections achieves comparable accuracy at a lower computational cost. Moreover, Grad-CAM ensures explainability, which is often lacking in transformer-based segmentation models.

Hybrid models, such as CoTr and UNETR, combine CNNs for local feature extraction with transformers for global context modeling, improving segmentation generalization across datasets. However, these models suffer from high training complexity and memory requirements, which limits their real-world feasibility. Moreover, interpretability remains an issue.

Enhancement in Our Approach: Our method provides a balance between CNN-based efficiency and interpretability, ensuring robust segmentation without high computational costs. The proposed method builds on these advancements by integrating Grad-CAM with a U-Net model specifically tailored for liver segmentation. Unlike previous works, this study evaluates the model’s robustness and explainability across multiple datasets acquired under varying conditions, ensuring both high performance and clinical applicability. By addressing gaps in the literature, such as the limited exploration of cross-dataset generalization and detailed Grad-CAM analyses, this research contributes a comprehensive and interpretable solution for liver segmentation.

The primary objective of the model is whole-liver segmentation, without explicitly distinguishing between normal hepatic parenchyma and pathological regions such as tumors, cysts, or transient hepatic attenuation differences (THADs). The missed lesions in Figure 5 and Figure 9 are likely due to several factors. First, the datasets used (DI and DII) focus on liver boundaries rather than tumor annotations, preventing the model from learning lesion-specific features. Additionally, many liver lesions exhibit low contrast on native CT scans, making them difficult to detect without arterial or venous phase enhancement. Finally, since the model was trained on single-phase CT images, it may struggle to generalize to lesions that appear differently across multiple imaging sequences. Given that lesion segmentation is critical for liver cancer diagnosis, the absence of this capability limits the direct clinical applicability of the model in oncology and hepatology.

To enhance the ability of the model to detect liver lesions, several improvements should be considered. One possible approach is incorporating multiphasic CT data by training the model on datasets such as LiTS, MSD, or 3D-IRCADb. This would allow the model to capture enhancement dynamics across arterial, venous, and delayed phases, thereby improving lesion segmentation accuracy. A multi-input deep learning model could be developed to fuse information from different phases, enabling a more comprehensive representation of liver lesions.

Another enhancement involves adapting the model for lesion segmentation. Instead of restricting the model to whole-liver segmentation, future iterations should incorporate multi-class segmentation that differentiates between normal liver tissue, benign lesions such as cysts, hemangiomas, THAD, and focal nodular hyperplasia (FNH), as well as malignant lesions including hepatocellular carcinoma (HCC), cholangiocarcinoma, and metastases. This extension could be implemented using a modified U-Net or a transformer-based segmentation model trained on datasets with annotated liver lesions.

Explainability is another crucial factor in improving lesion detection. Grad-CAM could be extended to highlight regions in the liver that contribute to an abnormal classification, providing radiologists with a visual representation of AI-based decisions. This would facilitate validation and enhance trust in automated segmentation results, making the model more interpretable and clinically applicable.

The proposed approach can seamlessly integrate into clinical workflows, assisting in preoperative planning, disease progression monitoring, and therapy assessment. Grad-CAM provides interpretability by highlighting areas of the image influencing the segmentation decisions. Improvements and explanations from the explainability point of view include the comparison of Grad-CAM outputs for correctly segmented and missegmented regions. It can highlight discrepancies to analyze failure cases and refine the model and to exploit Grad-CAM heatmaps to ensure that the highlighted regions correspond to liver edges and internal structures, confirming biologically plausible segmentation.

## 6. Conclusions and Future Work

Clinical imaging techniques, such as CT, currently represent essential tools that support clinicians in diagnosing diseases and planning appropriate interventions. This is especially crucial in malignant conditions like hepatocellular carcinoma, where precise image segmentation—specifically the delineation of the liver and tumor—serves as a critical first step in optimizing diagnosis, staging, and treatment strategies. Such treatments may include liver transplantation, surgical resection, radiotherapy, portal vein embolization, and various other procedures. Consequently, the effectiveness of segmentation methods can significantly impact both diagnostic accuracy and treatment outcomes.

In this paper, we proposed a deep learning-based approach to accurately segment the liver in medical images, addressing a pressing need in the diagnosis and treatment planning of hepatic diseases. Our approach leverages a U-Net architecture enhanced with residual connections, allowing for the capture of fine-grained anatomical details. We also prioritize prediction explainability by utilizing Grad-CAM, which highlights critical areas in the image that correspond to the segmentation results.

Experimental results reveal that the proposed method achieves an accuracy range of 0.81 to 0.93, demonstrating its potential as a reliable and efficient solution for automated liver segmentation. Moreover, we obtained a Dice index equal to 0.92 and a value related to IoU of 0.88.

As future work, we will consider different segmentation models, with the aim to try to improve the proposed performances. Moreover, we plan to adopt the proposed explainable segmentation method to such other organs as, for instance, the lung, with the aim of understanding whether it is also possible to provide explainable segmentation in other contexts. Furthermore, another interesting direction is to explore hybrid architectures that combine the strengths of convolutional neural networks and transformers. For instance, incorporating self-attention mechanisms within the U-Net framework could improve the model’s ability to capture long-range dependencies, especially when dealing with complex liver boundaries. Models like TransUNet, which integrate transformers into segmentation tasks, have shown promising results in other domains, and applying similar techniques to liver segmentation could enhance performance.

Although lesion segmentation is a crucial task, whole-liver segmentation remains valuable for defining the region of interest (ROI) in subsequent lesion detection and classification. It plays a significant role in preoperative planning, particularly for liver resection and transplantation. Additionally, volumetric assessment is essential for evaluating cirrhosis progression, steatosis, and hepatocellular carcinoma (HCC) treatment response. The proposed method ensures accurate delineation of liver boundaries, providing a strong foundation for lesion-specific segmentation in clinical applications.

To enhance the clinical applicability of this study, future work can focus on integrating lesion segmentation within the existing framework. One approach involves fine-tuning the model for tumor segmentation by incorporating annotated liver lesion datasets, such as those from the Liver Tumor Segmentation Challenge (LiTS). Adapting the current U-Net model to segment both normal liver tissue and pathological regions would enable a more comprehensive analysis. A multi-class segmentation strategy could also be implemented, allowing the network to distinguish between healthy parenchyma, benign lesions, and malignant tumors.

Beyond dataset expansion, incorporating attention mechanisms could improve lesion enhancement. While the current model achieves precise whole-liver segmentation, it may struggle to highlight small or low-contrast tumors. The addition of attention-based modules, such as Attention U-Net or Squeeze-and-Excitation (SE) blocks, would enable the model to focus on lesion-prone regions, improving sensitivity in detecting abnormalities. Furthermore, extending Grad-CAM explainability to highlight lesion areas that most influence the segmentation decision would enhance clinical trust by ensuring alignment between AI-based predictions and radiologists’ expectations.

The proposed method can also serve as a preprocessing step for lesion classification models. By integrating liver segmentation, lesion segmentation, and lesion classification, the workflow can evolve into a complete AI-driven diagnostic pipeline. This would facilitate tumor characterization, support treatment planning, and contribute to prognostic AI models for survival prediction. Combining segmentation with deep learning-based lesion classification and radiomics would provide a clinically meaningful framework for liver disease assessment.

While this study primarily focuses on whole-liver segmentation, future efforts will emphasize lesion differentiation to further improve its clinical relevance. Expanding the model to include liver lesion segmentation by leveraging publicly available annotated datasets will be a key objective. Additionally, attention-based mechanisms will be explored to enhance the detection of small or indistinct lesions. Finally, integrating lesion segmentation with classification models will contribute to the development of a more comprehensive AI-driven diagnostic workflow, improving the utility of automated liver analysis in clinical practice.

## Figures and Tables

**Table 1:** Hyperparameters of the proposed U-Net model.

Hyperparameter	Value
Model Architecture	U-Net with residual connections
Batch Size	16
Input Image Size	256 × 256 pixels
Optimizer	Adam
Initial Learning Rate	0.001
Learning Rate Decay	0.1 reduction every 100 epochs
Number of Epochs	500
Loss Function	Dice Loss + Cross-Entropy Loss
Activation Function	ReLU (Hidden Layers)/Sigmoid (Output Layer)s
Dropout Rate	0.3
Weight Initialization	He Normal
Augmentation Methods	Rotation (±20°), Flipping, Cropping, Gaussian Noise

**Table 2:** Comparison of deep learning-based liver segmentation methods.

Method	Backbone	DSC (%)
U-Net [30]	Encoder-Decoder	91.3
U-Net++ [35]	Dense U-Net	92.1
Attention U-Net [32]	Attention Mechanism	93.2
nnU-Net [36]	Self-Adaptive U-Net	94.6
DeepLabV3+ [37]	ResNet-101	92.5
TransUNet [38]	Transformer	93.8

## Data Availability

The original data presented in the study are openly available in Kaggle website and freely for research purposes (https://www.kaggle.com/datasets/, accessed on 27 February 2025).
